# Clinical significance of retained products of conception in placenta previa: a retrospective analysis

**DOI:** 10.1186/s12884-023-05805-0

**Published:** 2023-06-30

**Authors:** Naohisa Kishimoto, Morikazu Miyamoto, Akari Imauji, Minori Takada, Soko Nishitani, Risa Tanabe, Tsubasa Ito, Taira Hada, Yuka Otsuka, Masashi Takano

**Affiliations:** Department of Obstetrics and Gynecology, National Defense Medical College Hospital, 3-2, Namiki, Tokorozawa, Saitama 359-8513 Japan

**Keywords:** Retained products of conception, Placenta previa, Postpartum hemorrhage, Placenta accrete spectrum

## Abstract

**Background:**

Retained products of conception (RPOC) often cause severe postpartum hemorrhage (PPH) but the clinical significance of RPOC in placenta previa is unclear. This study aimed to investigate the clinical significance of RPOC in women with placenta previa. The primary outcome was to evaluate risk factors of RPOC and the secondary outcome was to consider risk factors of severe PPH.

**Methods:**

Singleton pregnant women with placenta previa who underwent cesarean section (CS) and placenta removal during the operation at the National Defense Medical College Hospital between January 2004 and December 2021 were identified. A retrospective analysis was performed to examine the frequency and risk factors of RPOC and the association of RPOC with severe PPH in pregnant women with placenta previa.

**Results:**

This study included 335 pregnant women. Among these, 24 (7.2%) pregnant women developed RPOC. Pregnant women with prior CS (Odds Ratio (OR) 5.98; 95% Confidence Interval (CI) 2.35–15.20,* p* < 0.01), major previa (OR 3.15; 95% CI 1.19–8.32, *p* < 0.01), and placenta accreta spectrum (PAS) (OR 92.7; 95% CI 18.39–467.22,* p* < 0.01) were more frequent in the RPOC group. Multivariate analysis revealed that prior CS (OR 10.70; 95% CI 3.47–33.00,* p* < 0.01,) and PAS (OR 140.32; 95% CI 23.84–825.79,* p* < 0.01) were risk factors for RPOC. In pregnant women who have placenta previa with RPOC or without RPOC, the ratio of severe PPH were 58.3% and 4.5%, respectively (*p* < 0.01). Furthermore, the occurrence of prior CS (OR 9.23; 95% CI 4.02–21.20,* p* < 0.01), major previa (OR 11.35; 95% CI 3.35–38.38,* p* < 0.01), placenta at the anterior wall (OR 3.44; 95% CI 1.40–8.44,* p* = 0.01), PAS (OR 16.47; 95% CI 4.66–58.26,* p* < 0.01), and RPOC (OR 29.70; 95% CI 11.23–78.55,* p* < 0.01) was more in pregnant women with severe PPH. In the multivariate analysis for severe PPH, prior CS (OR 4.71; 95% CI 1.29–17.13,* p* = 0.02), major previa (OR 7.50; 95% CI 1.98–28.43,* p* < 0.01), and RPOC (OR 13.26; 95% CI 3.61–48.63,* p* < 0.01) were identified as risk factors.

**Conclusions:**

Prior CS and PAS were identified as risk factors for RPOC in placenta previa and RPOC is closely associated with severe PPH. Therefore, a new strategy for RPOC in placenta previa is needed.

**Supplementary Information:**

The online version contains supplementary material available at 10.1186/s12884-023-05805-0.

## Background

Retained products of conception (RPOC) are defined as residual trophoblast-derived tissue that remains in the uterus after delivery, abortion, or miscarriage [1]. The overall frequency of the development of RPOC was reported to range from 1 to 6% [1–5]. The causes of RPOC have been identified as primipara, uterine atony, placenta accreta spectrum (PAS), history of RPOC, preterm delivery, prolonged use of oxytocin, previous uterine surgeries, and uterine abnormalities [1–5]. If not treated properly, patients with RPOC often develop severe postpartum hemorrhage (PPH) and endometritis [6, 7].

Placenta previa is defined as an abnormality of the placental location where placental parenchyma partially or completely covers the internal uterine ostium, which occasionally causes severe PPH [8–10]. Placenta previa is frequently complicated by PAS, which is considered a risk factor for RPOC [3]. Therefore, placenta previa may be closely associated with RPOC. Although both RPOC and placenta previa are associated with severe PPH [6, 8], the clinical significance of RPOC in placenta previa is unclear because there have been few reports on it [11, 12].

In this study, we retrospectively examined the incidence and risk factors of RPOC and investigated whether RPOC is related to severe PPH in patients with placenta previa.

## Methods

### Patient selection

Singleton pregnant women with placenta previa who underwent cesarean section (CS) and placenta removal during CS at the National Defense Medical College Hospital between January 2004 and December 2021 were identified. Patients without clinical information were excluded from the study.

**Fig. 1:**
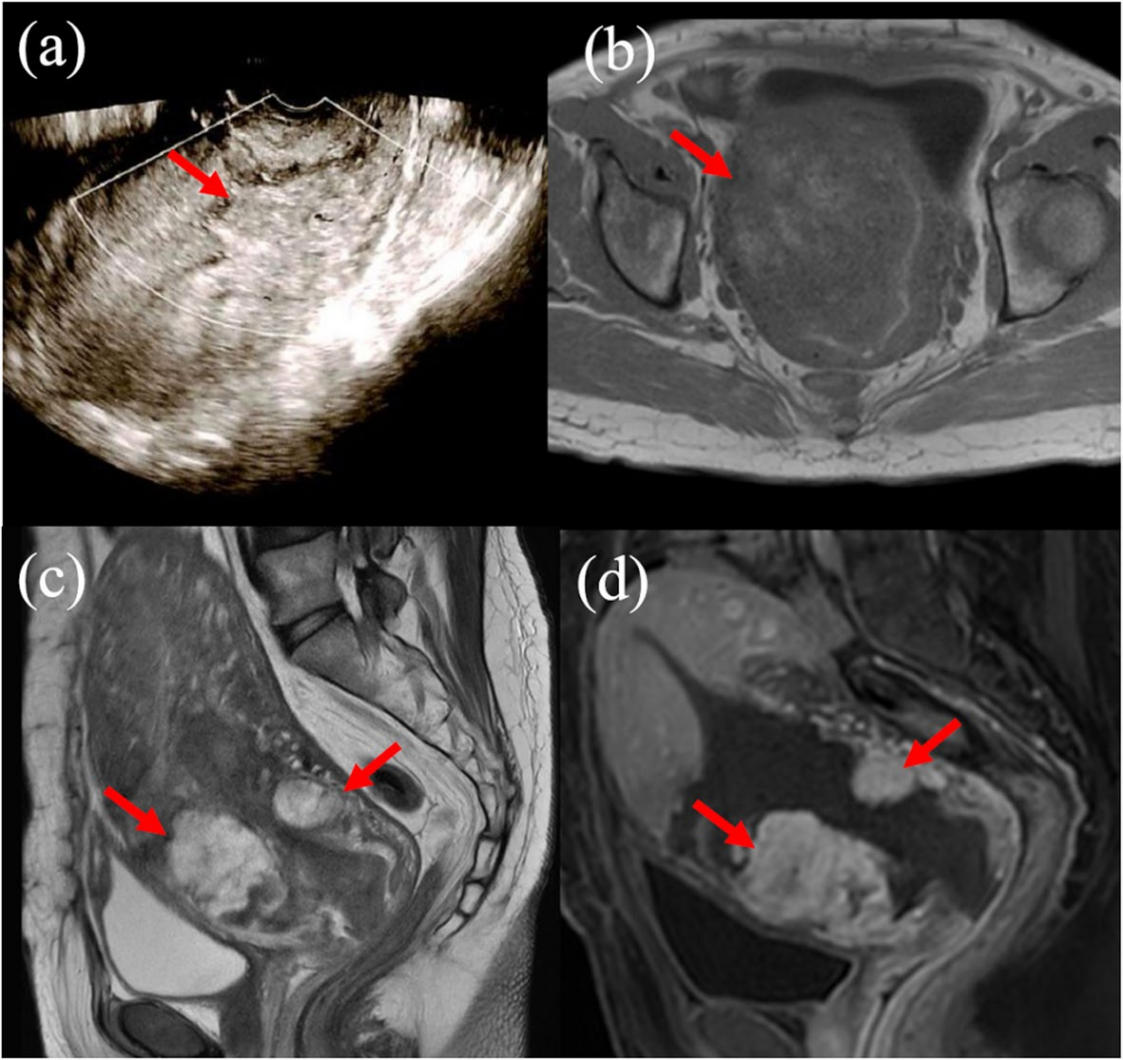
These images were diagnosed for retained products of conception (RPOC) in pregnant patients with placenta previa after operation. RPOC was represented as an echogenic mass at transvaginal color Doppler ultrasonography (US) after one day of operation (**a**), which was detected no flow, a heterogenous signal area at T1-weighted magnetic resonance imaging (MRI) (**b**), and a high signal area at T2-weighted MRI (**c**) and contrase-enhanced T1-weighted MRI (**d**) (red arrow heads show)

**Fig. 2:**
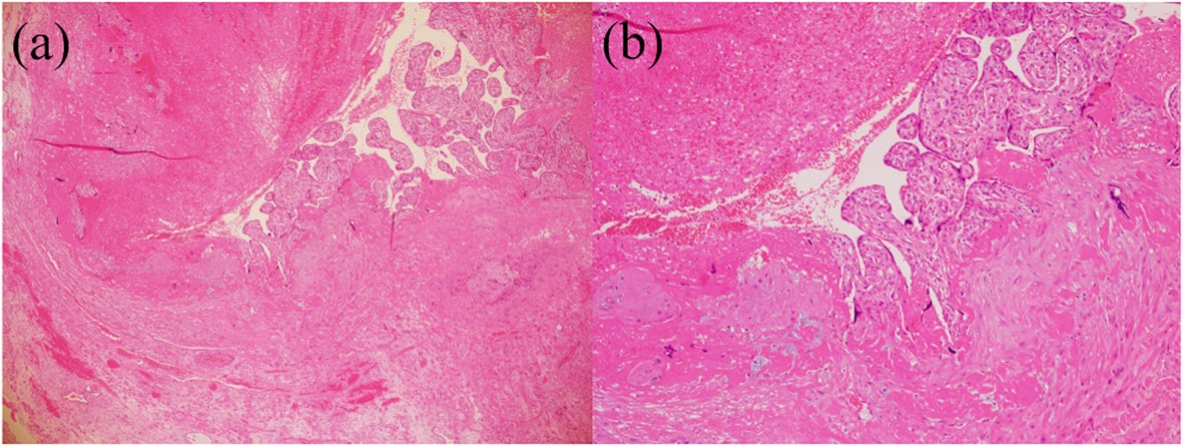
The pathological images of placenta accreta spectrum (PAS) in a pregnant patient with placenta previa. These images expressed a pathological feature of PAS; findings of invasion of trophoblastic tissue into the myometrium (red arrow heads show) and fibrin deposition in in some areas (blue arrow heads show). (**a** × 40, (**b**) × 100))

**Fig. 3:**
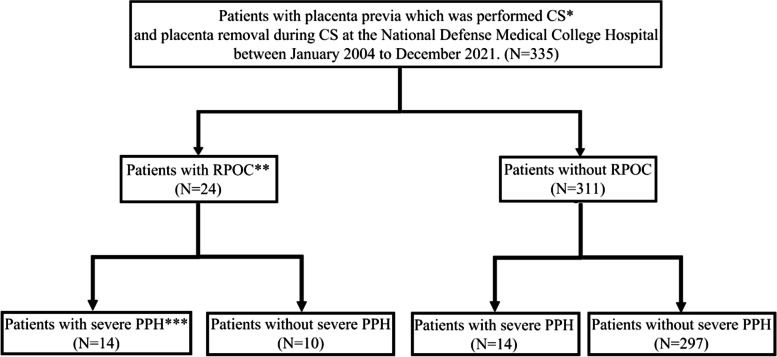
A study flow chart in pregnant patients with placenta previa. All patients with placenta previa were performed cesarean section (CS) (*). Retained products of conceptions (RPOC) (**) was diagnosed using ultrasonography (US), contrast-enhanced computed tomography (CT), and contrast-enhanced magnetic resonance imaging (MRI) according to previous reports until seven days after CS. Severe postpartum hemorrhage (severe PPH) (***) was defined as > 1000 ml of blood loss within 24 h of CS

RPOC was diagnosed using ultrasonography (US), contrast-enhanced computed tomography (CT), and contrast-enhanced magnetic resonance imaging (MRI) according to previous reports until seven days after CS [3, 6, 12–16]. When we suspected RPOC by US findings, intraoperatively and severe PPH, we implemented CT or MRI. Representative images of RPOC are shown in Fig. 1. A previous report classified placenta previa as major and minor previa [8, 17]. Cases of major previa were defined as those with a placenta that covered the internal cervical os, while cases with minor previa were those with the leading edge of the placenta, located within 2 cm from internal cervical OS, but did not cover the cervical os [8, 17–19]. PAS was finally diagnosed in all cases by pathological examination of the placenta and uterus (Fig. 2). When we suspected PAS intraoperatively, we used intravenous administration of uterotonic, insertion of uterine balloon tamponade, packing of sterile gauze into the vagina and compression suture during operation. If massive hemorrhage continued after operation, we considered UAE. When severe hemorrhage sustained after UAE, we might make a decision of hysterectomy [8, 20]. Severe PPH was defined as >1000 ml of blood loss within 24 h of CS [21–25]. Patients select flow chart showed at Fig. 3.

### Statistical analysis

The chi-square and Fisher’s exact tests were used to evaluate the clinicopathological features. Univariate and multivariate analyses were performed using logistic regression analysis. Multivariate analysis was performed using variables with statistical significance in univariate analysis. The level of statistical significance was set at *P* < 0.05.

## Results

During the observational period, 351 singleton pregnant women with placenta previa were identified, but 16 were excluded because of inadequate clinical information. Consequently, 335 patients were included in the analysis.

Among them, 24 (7.2%) were complicated by RPOC. In RPOC group, nine of 24 patients were suspected beforehand, sixteen of them intraoperatively, and seven both by beforehand and intraoperatively. Nine of 24 patients got pathological diagnosis of PAS, finally. Compared with pregnant women without RPOC, those with placenta previa and RPOC had a higher frequency of prior CS (*p* < 0.01), major previa (*p* < 0.01), and PAS (*p* < 0.01) (Table 1). Furthermore, they developed higher intraoperative blood loss (*p* < 0.01), postpartum hemorrhage (*p* < 0.01), and total blood loss (*p* < 0.01). Hence, all patients underwent US, and nine (2.7%) were diagnosed with RPOC.

**Table 1:** Characteristics of all pregnant women with placenta previa about retained products of concepts (RPOC)

	Pregnant women with RPOC		Pregnant women without RPOC		*p*-value
	*n* = 24		*n* = 311		
Maternal age					
> 35 years	14	(58.3%)	140	(45.0%)	0.29
< 35 years	10	(41.7%)	171	(55.0%)	
Gestational age at delivery					
> 37 weeks	14	(58.3%)	199	(64.0%)	0.66
< 37 weeks	10	(41.7%)	112	(36.0%)	
Parity					
Primipara	9	(37.5%)	161	(51.8%)	0.21
Multipara	15	(62.5%)	150	(48.2%)	
In vitro fertilization pregnancy					
Yes	5	(20.8%)	38	(12.2%)	0.21
No	19	(79.2%)	273	(87.8%)	
Tocolytic agent use					
Yes	14	(58.3%)	124	(39.9%)	0.09
No	10	(41.7%)	187	(60.1%)	
Warning bleeding^a^					
Yes	11	(45.8%)	86	(27.7%)	0.07
No	13	(54.2%)	225	(72.3%)	
Prior cesarean section					
Yes	11	(45.8%)	33	(10.6%)	< 0.01
No	13	(54.2%)	278	(89.4%)	
The mode of cesarean section					
Emergency	6	(25.0%)	67	(21.5%)	0.80
Elective	18	(75.0%)	244	(78.5%)	
The classification of placenta previa					
major previa^b^	18	(75.0%)	137	(44.0%)	< 0.01
minor previa^c^	6	(25.0%)	174	(56.0%)	
Main location of placenta					
Anterior wall	6	(25.0%)	34	(10.9%)	0.052
Posterior wall	18	(75.0%)	277	(89.1%)	
Placenta accreta spectrum					
Yes	9	(37.5%)	2	(0.6%)	< 0.01
No	15	(62.5%)	309	(99.4%)	
Prior endometrial curettage					
Yes	8	(33.3%)	83	(26.7%)	0.48
No	16	(66.7%)	228	(73.3%)	
Magnetic resonance imaging after operation					
Yes	14	(58.3%)	14	(4.5%)	< 0.01
No	10	(41.7%)	297	(95.5%)	
Intraoperative blood loss (ml)					
median (range)	1849	(724–4936)	1119	(265–3418)	< 0.01
Postpartum hemorrhage (ml)					
median (range)	1868	(20–8748)	120	(0–3261)	< 0.01
Total blood loss (ml)					
median (range)	4025	(789–13,172)	1270	(287–5121)	< 0.01

^a^Warning bleeding was defined as painless genital bleeding from the placenta

^b^Major previa was defined as a placenta that covered the internal cervical os

^c^Minor previa was defined as the leading edge of the placenta, which was located within 2 cm from internal cervical OS but did not cover the cervical os

We suspected RPOC in 32 of 335 (9.6%) patients with placenta previa by intraoperative findings or severe PPH, and we were performed by CT in one of them and MRI in 31 of them, following ultrasound examination. The woman who received CT and 14 out of 31 (45.1%) pregnant women who underwent MRI were diagnosed with RPOC.

In the univariate analysis, prior CS (odds ratio (OR), 7.13; *p* < 0.01), major previa (OR, 3.86; *p* < 0.01), placenta at the anterior wall (OR, 2.72; *p* = 0.048), and PAS (OR, 92.7; *p* < 0.01) were identified. Multivariate analysis revealed that prior CS (odds ratio [OR] 10.70; *p* < 0.01) and PAS (OR 140.32; *p* < 0.01) were independent risk factors for RPOC (Table 2).

**Table 2:** Univariate and multivariate analysis to investigate the risk factor of retained products of conception in pregnant women with placenta previa

	Univariate analysis			Multivariate analysis^d^		
	Odds ratio (95% Confidence interval)		*p*-value	Odds ratio (95% Confidence interval)		*p*-value
Maternal age						
> 35 years vs < 35 years	1.68	(0.73–3.92)	0.22			
Gestational age at delivery						
> 37 weeks vs < 37 weeks	1.27	(0.55–2.95)	0.58			
Parity						
Primipara vs Multipara	0.57	(0.24–1.33)	0.19			
In vitro fertilization pregnancy						
Yes vs. No	1.84	(0.65–5.20)	0.25			
Tocolytic agent use						
Yes vs. No	2.08	(0.90–4.84)	0.09			
Warning bleeding^a^						
Yes vs. No	2.21	(0.96–5.13)	0.06			
Prior cesarean section						
Yes vs. No	7.13	(2.96–17.19)	< 0.01	10.70	(3.47–33.00)	< 0.01
The mode of cesarean section						
Emergency vs Elective	1.21	(0.46–3.18)	0.69			
The classification of placenta previa						
Major previa^b^ vs Minor previa^c^	3.86	(1.49–9.99)	< 0.01	1.72	(0.55–5.42)	0.35
Main location of placenta						
Anterior wall vs Posterior wall	2.72	(1.01–7.32)	0.048	1.38	(0.37–5.7)	0.63
Placenta accreta spectrum						
Yes vs. No	92.7	(18.39–467.22)	< 0.01	140.32	(23.84–825.79)	< 0.01
Prior endometrial curettage						
Yes vs. No	1.37	(0.57–3.33)	0.48			

^a^Warning bleeding was defined ass painless genital bleeding from the placenta

^b^Major previa was defined as a placenta that covered the internal cervical os

^c^Minor previa was defined as the leading edge of the placenta, which was located within 2 cm from internal cervical OS but did not cover the cervical os

^d^Multivariate analysis was adjusted for prior cesarean section, the classification of placenta previa, main location of placenta, and placenta accreta spectrum

Of these, 28 (8.4%) pregnant women with placenta previa developed severe PPH. The proportion of pregnant women with placenta previa and severe PPH who experienced multipara (p = 0.048), prior CS (*p* < 0.01), major previa (*p* < 0.01), placenta at the anterior wall (*p* = 0.01), PAS (*p* < 0.01), and RPOC (*p* < 0.01) was more than those with placenta previa without severe PPH. Ten patients with RPOC had no severe PPH. When we performed them by routine US before leaving hospital, they were diagnosed by chance. (Table 3). In univariate analysis, multiparity (OR 0.43; *p* = 0.04), prior CS (OR 9.23; *p* < 0.01), major previa (OR 11.35; *p* < 0.01), placenta at the anterior wall (OR 3.44; *p* < 0.01), PAS (OR 16.47; *p* < 0.01), and RPOC (OR 29.70; *p* < 0.01) were identified as risk factors for severe PPH. Multivariate analysis showed that prior CS (OR, 4.71; *p* < 0.01), major previa (OR, 7.50; *p* < 0.01), and RPOC (OR, 13.26; *p* < 0.01) were independent risk factors for severe PPH (Table 4).

**Table 3:** Characteristics of all pregnant women with placenta previa according to the amount of the postpartum hemorrhage (PPH)

	Pregnant patients with severe PPH^a^		Pregnant patients without severe PPH		*p*-value
	*n* = 28		*n* = 307		
Maternal age					
> 35 years	14	(50.0%)	140	(45.6%)	0.70
< 35 years	14	(50.0%)	167	(54.4%)	
Gestational age at delivery					
> 37 weeks	18	(64.3%)	195	(63.5%)	1.00
< 37 weeks	10	(35.7%)	112	(36.5%)	
Parity					
Primipara	9	(32.1%)	161	(52.6%)	0.048
Multipara	19	(67.9%)	146	(47.4%)	
In vitro fertilization pregnancy					
Yes	3	(10.7%)	40	(13.0%)	1.00
No	25	(89.3%)	267	(87.0%)	
Tocolytic agent use					
Yes	13	(46.4%)	125	(40.7%)	0.55
No	15	(53.6%)	182	(59.3%)	
Warning bleeding^b^					
Yes	10	(35.7%)	87	(28.3%)	0.39
No	18	(64.3%)	220	(71.7%)	
Prior CS					
Yes	14	(30.0%)	30	(9.8%)	< 0.01
No	14	(0.0%)	277	(90.2%)	
The mode of cesarean section					
Emergency	6	(21.4%)	67	(21.8%)	1.00
Elective	22	(78.6%)	240	(78.2%)	
The classification of placenta previa					
Major previa^c^	25	(89.3%)	130	(42.4%)	< 0.01
Minor previa^d^	3	(10.7%)	177	(57.6%)	
Main location of placenta					
Anterior wall	8	(28.6%)	32	(10.4%)	0.01
Posterior wall	20	(71.4%)	275	(89.6%)	
Placenta accreta spectrum					
Yes	6	(21.4%)	5	(1.6%)	< 0.01
No	22	(78.6%)	302	(98.4%)	
Prior endometrial curettage					
Yes	6	(21.4%)	85	(27.7%)	0.66
No	22	(78.6%)	222	(72.3%)	
Retained products of conception					
Yes	14	(50.0%)	10	(3.3%)	< 0.01
No	14	(50.0%)	297	(96.7%)	

*PPH *postpartum hemorrhage, *CS *cesarean section

^a^Severe PPH was defined as > 1000 ml of blood loss within 24 h of CS

^b^Warning bleeding was defined as painless genital bleeding from the placenta

^c^Major previa was defined as the placenta that covered the internal cervical os

^d^Minor previa was defined as the leading edge of the placenta, which was located within 2 cm from internal cervical OS but did not cover the cervical os

**Table 4:** Univariate and multivariate analysis to examine the risk factor of severe PPH^a^ in pregnant women with placenta previa

	Univariate analysis			Multivariate analysis		
	Odds ratio (95% Confidence interval)		*p*-value	Odds ratio (95% Confidence interval)		*p*-value
Maternal age						
> 35 years vs. < 35 years	1.19	(0.55–2.59)	0.66			
Gestational age at delivery						
> 37 weeks vs. < 37 weeks	1.03	(0.46–2.32)	0.94			
Parity						
Primipara vs Multipara	0.43	(0.19–0.98)	0.04	0.95	(0.27–3.33)	0.93
In vitro fertilization pregnancy						
Yes vs. No	0.80	(0.23–2.78)	0.73			
Tocolytic agent use						
Yes vs. No	1.26	(0.58–2.74)	0.56			
Warning bleeding^b^						
Yes vs. No	1.40	(0.62–3.16)	0.41			
Prior CS						
Yes vs. No	9.23	(4.02–21.20)	< 0.01	4.71	(1.29–17.13)	0.02
The mode of cesarean section						
Emergency vs. Elective	0.98	(0.38–2.51)	0.96			
The classification of placenta previa						
Major previa^c^ vs. Minor previa^d^	11.35	(3.35–38.38)	< 0.01	7.50	(1.98–28.43)	< 0.01
Main location of placenta						
Anterior wall vs. Posterior wall	3.44	(1.40–8.44)	< 0.01	1.83	(0.56–6.00)	0.32
Placenta accreta spectrum						
Yes vs. No	16.47	(4.66–58.26)	< 0.01	2.53	(0.39–16.46)	0.33
Prior endometrial curettage						
Yes vs. No	0.71	(0.28–1.82)	0.48			
Retained products of conception						
Yes vs. No	29.70	(11.23–78.55)	< 0.01	13.26	(3.61–48.63)	< 0.01

*PPH *postpartum hemorrhage, *CS *cesarean section

^a^Severe PPH was defined as > 1000 ml of blood loss within 24 h of CS

^b^Warning bleeding was defined as painless genital bleeding from the placenta

^c^Major previa was defined as the placenta that covered the internal cervical os

^d^Minor previa was defined as the leading edge of the placenta, which was located within 2 cm from internal cervical OS but did not cover the cervical os

Details of the 14 cases of RPOC that developed severe PPH show in supplementary material 1. Thirteen (92.9%) cases required additional allogeneic blood transfusions. Nine (64.2%) cases needed uterine artery embolization (UAE) after CS. Within 11 weeks after CS, dilation and curettage (D&C) were performed to remove placenta in three (21.4%) cases. Ten (71.4%) patients received intrauterine balloon tamponade to decrease the amount of blood loss after CS. Two (14.3%) patients required supravaginal amputation of the uterus and total hysterectomy as UAE and intrauterine balloon tamponade could not stop bleeding after CS. Thirteen (92.9%) patients with RPOC who developed severe PPH had major previa.

Details of the 10 pregnant women with RPOC without severe PPH show in supplementary material 2. Two (20%) of them required additional allogeneic blood transfusion. Six (60%) cases underwent intrauterine balloon tamponade to decrease the amount of blood loss after CS. One patient required D&C 11 weeks after CS. Three weeks after CS, one (10%) patient developed > 2500 ml of blood loss and required UAE to stop bleeding. Subsequently, the RPOC reduced naturally.

Four (17%) of 24 pregnant women with RPOC needed D&C, and 10 (42%) required UAE. Two (8%) of the 24 had hysterectomy for RPOC treatment. Finally, in 13 (54%) pregnant women, RPOC was reduced naturally without D&C, UAE, or hysterectomy treatment.

## Discussion

In this study, we found that pregnant women with placenta previa with RPOC experienced prior CS complicated with major previa, PAS, developed massive intraoperative blood loss, massive postpartum hemorrhage, and massive total blood loss more frequently than those without RPOC did. Multivariate analysis revealed that prior CS and PAS were the risk factors for RPOC in pregnant women with placenta previa. Pregnant women with placenta previa with severe PPH experienced multipara, prior CS, major previa, placenta at the anterior wall, PAS, and RPOC more frequently than pregnant women having placenta previa without severe PPH. In addition, multivariate analysis demonstrated that RPOC was the cause of severe PPH in addition to prior CS and major previa. Many pregnant women with RPOC receive several additional hemostatic treatments.

In previous studies, risk factors for RPOC were primipara, uterine atony, placenta accreta spectrum (PAS), history of RPOC, preterm delivery, prolonged oxytocin intake, previous uterine surgeries, and uterine abnormalities [1–5]. This study identified prior CS and PAS as risk factors for RPOC in women with placenta previa. Therefore, our findings partially correspond to previous reports, and we need to pay attention to RPOC in pregnant women with placenta previa who have prior CS and suspicious PAS.

In previous studies, the incidence of the development of RPOC was reported to range from 1 to 6% [1–5]. Hence, in this study, there was a possibility that the occurrence rate of RPOC in placenta previa (7.2%) was higher, although a direct comparison is not feasible. This discrepancy was assumed to cause differences in subjects between previous reports and our study. Although previous reports have included women after delivery, abortion, or miscarriage, our study included all pregnant women with placenta previa. Therefore, pregnant women with placenta previa often develop RPOC.

The tools for the diagnosis of RPOC were US, CT, and MRI [3, 6, 12–16]. Of these, US is frequently used with a sensitivity and specificity ranging from 66 to 98% and 33 to 89%, respectively [4, 26–28]. CT and MRI are assistive tools to diagnose RPOC, but their sensitivities and specificities are unclear due to the small number of case studies [3, 6]. In our study, some cases could not be diagnosed by US, but were diagnosed with MRI and CT. Therefore, MRI and CT are useful tools for diagnosing RPOC, and it may be valuable that all patients with placenta previa will be performed by CT and MRI to detect RPOC. On the other hand, previous studies revealed that age, prior D&C, prior CS, major previa, and anterior locating placenta were the risk factors for severe PPH in placenta previa [29, 30]. In our study, RPOC was considered a risk factor for severe PPH in pregnant women with placenta previa. Since RPOC might induce massive PPH, diagnosis using MRI or CT may be actively recommended. But few previous studies revealed the sensitivity and the specificity of CT or MRI to diagnose RPOC, and we might miss patients who had RPOC without severe PPH because of a difference of modalities. Further studies are needed to discover an availability of CT and MRI for diagnosis of RPOC in all patients with placenta previa after operation.

Most cases of RPOC received no treatment because RPOC disappeared naturally, with its incidence ranging from 55 to 71% [14, 31]. Hence, in the case that patients underwent any treatment, many pregnant women with RPOC received D&C and recovered [31, 32]. However, although the incidence was low, if massive PPH developed, UAE and hysterectomy were performed, and massive blood transfusion was required [12, 31–34]. In this study, many pregnant women with placenta previa with RPOC underwent insertion of uterine balloon tamponade, UAE, hysterectomy, and massive blood transfusion, although there were patients who received no treatment. Therefore, RPOC is a more important factor that causes massive hemorrhage in patients with placenta previa than in those without placenta previa. In previous studies, few patients required UAE or hysterectomy, but this study revealed that more patients with placenta previa required UAE or hysterectomy. Therefore, the clinical significance of RPOC in placenta previa should be recognized. Previous studies showed that insertion of uterine balloon tamponade decreased the intraoperative and postoperative hemorrhage in patients with placenta previa [8, 35]. In this study, patients with placenta previa who received RPOC could benefit insertion of uterine balloon tamponade and it might decrease postpartum hemorrhage. But the number of cases was small. The Further study is needed and we should evaluate a new strategy that we will routinely insert of uterine balloon tamponade in all patients with RPOC in order to decrease postpartum hemorrhage.

In our hospital, all patients were performed ultrasound examination after operation within seven days because placental remnants are empirically frequent. All cases who had severe PPH were performed by US within 24 h after operation as a rule of our hospital to search the cause of severe PPH. Therefore, all patients had at least one ultrasound examination performed during stay in our hospital.

The present study had some limitations. First, this was a retrospective, single-institutional study and included only a small sample size. Second, we did not perform a literature review or meta-analysis and could not evaluate the further risk factor. Third, patients with severe PPH were decreased because almost all patients had inserted uterine balloon tamponade after operation. Finally, at inclusion criteria in this study, we targeted at only singleton pregnant women with placenta previa who underwent CS and placenta removal during CS. Therefore, patients with PAS who had needed UAE and hysterectomy might be decreased. However, our study demonstrated the clinical significance of RPOC and is useful in clinical settings and future studies.

## Conclusion

Prior CS and PAS are risk factors for RPOC development. In addition, RPOC may be a risk factor for massive PPH in placenta previa. Therefore, the development of a definitive treatment is necessary.

## Supplementary Information

**Additional file 1.** **Supplementary material 1.** The details of cases with retained products of conception (RPOC)with severe postpartum hemorrhage in pregnant patients with placenta previa.

**Additional file 2.** **Supplementary material 2.** The details of cases with retained products of conception (RPOC)without severe postpartum hemorrhage in pregnant patients with placenta previa.

## Data Availability

The datasets used and/or analysed during the current study are available from the corresponding author on reasonable request.

## Abbreviations

RPOC: Retained products of conception
PPH: Postpartum hemorrhage
CS: Cesarean section
PAS: Placenta accreta spectrum
OR: Odds ratio
US: Ultrasonography
MRI: Magnetic resonance imaging
CT: Contrast-enhanced computed tomography
UAE: Uterine artery embolization
D&C: Dilation and curettage

## References

[1] Romero R, Hsu YC, Athanassiadis AP, Hagay Z, Avila C, Nores J (1990). Preterm delivery: a risk factor for retained placenta. Am J Obstet Gynecol.

[2] Hoveyda F, MacKenzie IZ (2001). Secondary postpartum haemorrhage: incidence, morbidity and current management. BJOG.

[3] Sellmyer MA, Desser TS, Maturen KE, Jeffrey RB, Kamaya A (2013). Physiologic, histologic, and imaging features of retained products of conception. Radiographics.

[4] Van den Bosch T, Daemen A, Van Schoubroeck D, Pochet N, De Moor B (2008). Timmerman D (2008) Occurrence and outcome of residual trophoblastic tissue: a prospective study. J Ultrasound Med.

[5] Ganer Herman H, Kogan Z, Tairy D, Ben Zvi M, Kerner R, Ginath S (2018). Pregnancies following hysteroscopic removal of retained products of conception after delivery versus abortion. Gynecol Obstet Invest.

[6] Iraha Y, Okada M, Toguchi M, Azama K, Mekaru K, Kinjo T (2018). Multimodality imaging in secondary postpartum or postabortion hemorrhage: retained products of conception and related conditions. Jpn J Radiol.

[7] Abbasi S, Jamal A, Eslamian L, Marsousi V (2008). Role of clinical and ultrasound findings in the diagnosis of retained products of conception. Ultrasound Obstet Gynecol.

[8] Soyama H, Miyamoto M, Ishibashi H, Nakatsuka M, Kawauchi H, Sakamoto T (2019). Analysis of prophylactic Bakri balloon tamponade failure in patients with placenta previa. Taiwan J Obstet Gynecol.

[9] van Wessel S, Coryn N, van Vliet H, Schoot B, Weyers S, Hamerlynck T (2020). Reproductive and obstetric outcomes after hysteroscopic removal of retained products of conception. J Minim Invasive Gynecol.

[10] Jauniaux E, Alfirevic Z, Bhide AG, Belfort MA, Burton GJ, Collins SL (2019). Placenta praevia and placenta accreta: diagnosis and management. BJOG: Int J Obstet Gy: green-top guideline.L. BJOG.

[11] Silver RM, Branch DW (2018). Placenta accreta spectrum. N Engl J Med.

[12] Vyas S, Choi HH, Whetstone S, Jha P, Poder L, Shum DJ (2021). Ultrasound features help identify patients who can undergo noninvasive management for suspected retained products of conception: a single institutional experience. Abdom Radiol (NY).

[13] Tanimura K, Yamasaki Y, Ebina Y, Deguchi M, Ueno Y, Kitajima K (2015). Prediction of adherent placenta in pregnancy with placenta previa using ultrasonography and magnetic resonance imaging. Eur J Obstet Gynecol Reprod Biol.

[14] Ou J, Peng P, Teng L, Li C, Liu X (2019). Management of patients with placenta accreta spectrum disorders who underwent pregnancy terminations in the second trimester: a retrospective study. Eur J Obstet Gynecol Reprod Biol.

[15] Groszmann YS, Healy Murphy AL, Benacerraf BR (2018). Diagnosis and management of patients with enhanced myometrial vascularity associated with retained products of conception. Ultrasound Obstet Gynecol.

[16] Hamel CC, van Wessel S, Carnegy A, Coppus SFPJ, Snijders MPML, Clark J (2021). Diagnostic criteria for retained products of conception-A scoping review. Acta Obstet Gynecol Scand.

[17] Calì G, Giambanco L, Puccio G, Forlani F (2013). Morbidly adherent placenta: evaluation of ultrasound diagnostic criteria and differentiation of placenta accreta from percreta. Ultrasound Obstet Gynecol.

[18] Grönvall M, Stefanovic V, Paavonen J, Loukovaara M, Tikkanen M (2019). Major or minor placenta previa: does it make a difference?. Placenta.

[19] Bahar A, Abusham A, Eskandar M, Sobande A, Alsunaidi M (2009). Risk factors and pregnancy outcome in different types of placenta previa. J Obstet Gynaecol Can.

[20] Van Wyck HB (1943). Antepartum Haemorrhage. Can Med Assoc J.

[21] WHO guidelines for the management of postpartum haemorrhage and retained placenta. https://apps.who.int/iris/handle/10665/44171. Accessed 04 Nov 2022. 23844453

[22] Prevention and Management of Postpartum Haemorrhage: Green-top Guideline No. 52. BJOG. 2017;124(5):e106–49. 10.1111/1471-0528.14178 27981719

[23] Franke D, Zepf J, Burkhardt T, Stein P, Zimmermann R, Haslinger C (2021). Retained placenta and postpartum hemorrhage: time is not everything. Arch Gynecol Obstet.

[24] Liu CN, Yu FB, Xu YZ, Li JS, Guan ZH, Sun MN (2021). Prevalence and risk factors of severe postpartum hemorrhage: a retrospective cohort study. BMC Pregnancy Childbirth.

[25] Neary C, Naheed S, McLernon DJ, Black M (2021). Predicting risk of postpartum haemorrhage: a systematic review. BJOG.

[26] Durfee SM, Frates MC, Luong A, Benson CB (2005). The sonographic and color Doppler features of retained products of conception. J Ultrasound Med.

[27] Matijevic R, Knezevic M, Grgic O, Zlodi-Hrsak L (2009). Diagnostic accuracy of sonographic and clinical parameters in the prediction of retained products of conception. J Ultrasound Med.

[28] de Vries JI, van der Linden RM, van der Linden HC (2000). Predictive value of sonographic examination to visualize retained placenta directly after birth at 16 to 28 weeks. J Ultrasound Med.

[29] Kong CW, To WWK (2020). Risk factors for severe postpartum haemorrhage during caesarean section for placenta praevia. J Obstet Gynaecol.

[30] Pivano A, Alessandrini M, Desbriere R, Agostini A, Opinel P, d’Ercole C (2015). A score to predict the risk of emergency caesarean delivery in women with antepartum bleeding and placenta praevia. Eur J Obstet Gynecol Reprod Biol.

[31] Kamaya A, Krishnarao PM, Nayak N, Jeffrey RB, Maturen KE (2016). Clinical and imaging predictors of management in retained products of conception. Abdom Radiol (NY).

[32] Takahashi H, Ohhashi M, Baba Y, Nagayama S, Ogoyama M, Horie K (2019). Conservative management of retained products of conception in the normal placental position: a retrospective observational study. Eur J Obstet Gynecol Reprod Biol.

[33] Shitanaka S, Chigusa Y, Kawahara S, Kawasaki K, Mogami H, Mandai M, Kondoh E (2020). Conservative management for retained products of conception after less than 22 weeks of gestation. J Obstet Gynaecol Res.

[34] Kimura Y, Osuga K, Nagai K, Hongyo H, Tanaka K, Ono Y (2020). The efficacy of uterine artery embolization with gelatin sponge for retained products of conception with bleeding and future pregnancy outcomes. CVIR Endovasc.

[35] Soyama H, Miyamoto M, Sasa H, Ishibashi H, Yoshida M, Nakatsuka M, Takano M, Furuya K (2017). Effect of routine rapid insertion of Bakri balloon tamponade on reducing hemorrhage from placenta previa during and after cesarean section. Arch Gynecol Obstet.

