# Supplementary material for: Clinical significance of retained products of conception in placenta previa: a retrospective analysis
Source: BMC Pregnancy Childbirth. 2023 Jun 30;23:481. doi: 10.1186/s12884-023-05805-0 (PMC10311830; doi:10.1186/s12884-023-05805-0)
Supplement: Supplementary file 1 — Additional file 1. Supplementary material 1. The details of cases with retained products of conception (RPOC)with severe postpartum hemorrhage in pregnant patients with placenta previa. [file 12884_2023_5805_MOESM1_ESM.docx]

Supplementary material 1 The details of cases with retained products of conception (RPOC) with severe postpartum hemorrhage in pregnant patients with placenta previa.

| No* | Age | Prior cesarean section | The classification of placenta previa | Main location of placenta | Placenta accrete  spectrum | Intraoperative blood loss (ml) | The amount of postpartum hemorrhage(ml) | Treatment for RPOC |
| --- | --- | --- | --- | --- | --- | --- | --- | --- |
| 1 | 37 | No | Major previa* | Posterior wall | Yes | 4627 | 2307 | Allogeneic blood transfusion  Uterine artery embolization (UAE)  Dilation and curettage (D&C) 11 weeks after cesarean section (CS). |
| 2 | 40 | Yes | Major previa* | Posterior wall | Yes | 1649 | 3253 | Allogeneic blood transfusion  UAE  D&C at 6 weeks after CS |
| 3 | 31 | No | Major previa* | Posterior wall | Yes | 2774 | 3253 | Allogeneic blood transfusion  UAE |
| 4 | 32 | Yes | Major previa* | Anterior wall | No | 4936 | 6386 | Allogeneic blood transfusion  UAE  D&C one week after CS. |
| 5 | 31 | Yes | Major previa* | Posterior wall | No | 770 | 3050 | Allogeneic blood transfusion  UAE  Intrauterine balloon tamponade |
| 6 | 36 | No | Minor previa** | Posterior wall | No | 3945 | 3335 | Allogeneic blood transfusion  UAE  Intrauterine balloon tamponade |
| 7 | 39 | No | Major previa* | Posterior wall | No | 1202 | 3028 | Allogeneic blood transfusion  Intrauterine balloon tamponade |
| 8 | 34 | Yes | Major previa* | Anterior wall | No | 938 | 4345 | Allogeneic blood transfusion  Intrauterine balloon tamponade |
| 9 | 34 | Yes | Major previa* | Posterior wall | No | 3264 | 1430 | Allogeneic blood transfusion  Intrauterine balloon tamponade |
| 10 | 30 | No | Major previa* | Anterior wall | Yes | 1354 | 5311 | Allogeneic blood transfusion  UAE  Intrauterine balloon tamponade |
| 11 | 34 | Yes | Major previa* | Posterior wall | No | 2139 | 2336 | Allogeneic blood transfusion  Intrauterine balloon tamponade |
| 12 | 40 | No | Major previa* | Posterior wall | No | 2043 | 1127 | Intrauterine balloon tamponade |
| 13 | 34 | Yes | Major previa* | Anterior wall | Yes | 4424 | 8748 | Allogeneic blood transfusion  UAE  Intrauterine balloon tamponade  Supravaginal amputation of the uterus at one day after CS. |
| 14 | 39 | No | Major previa* | Anterior wall | Yes | 4240 | 7488 | Allogeneic blood transfusion  UAE  Intrauterine balloon tamponade  Total hysterectomy at the day of CS. |

*Major previa was defined as a placenta that covered the internal cervical os.

**Minor previa was defined as the leading edge of the placenta located within 2cm from internal cervical OS but did not cover the cervical os.
