# Supplementary material for: Clinical significance of retained products of conception in placenta previa: a retrospective analysis
Source: BMC Pregnancy Childbirth. 2023 Jun 30;23:481. doi: 10.1186/s12884-023-05805-0 (PMC10311830; doi:10.1186/s12884-023-05805-0)
Supplement: Supplementary file 2 — Additional file 2. Supplementary material 2. The details of cases with retained products of conception (RPOC)without severe postpartum hemorrhage in pregnant patients with placenta previa. [file 12884_2023_5805_MOESM2_ESM.docx]

Supplementary material 2 The details of cases with retained products of conception (RPOC) without severe postpartum hemorrhage in pregnant patients with placenta previa.

| No* | Age | Prior cesarean section | The classification of placenta previa | Main location of placenta | Placenta accrete  spectrum | Intraoperative blood loss (ml) | The amount of postpartum hemorrhage(ml) | Treatment for RPOC |
| --- | --- | --- | --- | --- | --- | --- | --- | --- |
| 15 | 41 | No | Minor previa** | Posterior wall | Yes | 1419 | 375 | Observation |
| 16 | 33 | Yes | Major previa* | Posterior wall | No | 1943 | 204 | Allogeneic blood transfusion |
| 17 | 38 | No | Major previa* | Posterior wall | No | 1526 | 389 | Dilation and curettage at 11 weeks after operation |
| 18 | 43 | Yes | Minor previa** | Posterior wall | No | 1190 | 45 | Observation |
| 19 | 41 | No | Minor previa** | Posterior wall | Yes | 724 | 65 | Intrauterine balloon tamponade |
| 20 | 39 | No | Major previa* | Posterior wall | Yes | 1562 | 112 | Intrauterine balloon tamponade  UAE 3 weeks after caesarian section because of 2500 ml blood loss |
| 21 | 39 | No | Major previa* | Posterior wall | No | 901 | 20 | Intrauterine balloon tamponade |
| 22 | 31 | Yes | Major previa* | Posterior wall | No | 1777 | 694 | Intrauterine balloon tamponade |
| 23 | 40 | No | Minor previa** | Anterior wall | No | 1922 | 916 | Allogeneic blood transfusion  Intrauterine balloon tamponade |
| 24 | 41 | Yes | Minor previa** | Posterior wall | No | 2096 | 20 | Intrauterine balloon tamponade |

*Major previa was defined as a placenta that covered the internal cervical os.

**Minor previa was defined as the leading edge of the placenta, which was located within 2cm from internal cervical OS but did not cover the cervical os.
